# Modified Ravitch procedure for pectus excavatum in Marfan syndrome with annuloaortic ectasia

**DOI:** 10.1093/icvts/ivad095

**Published:** 2023-06-12

**Authors:** Takahiro Iida, Kazuhiro Nagayama, Kentaro Kitano, Haruo Yamauchi, Minoru Ono, Jun Nakajima

**Affiliations:** Department of Thoracic Surgery, The University of Tokyo Graduate School of Medicine, Tokyo, Japan; Department of Thoracic Surgery, The University of Tokyo Graduate School of Medicine, Tokyo, Japan; Department of Thoracic Surgery, The University of Tokyo Graduate School of Medicine, Tokyo, Japan; Department of Cardiac Surgery, The University of Tokyo Graduate School of Medicine, Tokyo, Japan; Department of Cardiac Surgery, The University of Tokyo Graduate School of Medicine, Tokyo, Japan; Department of Thoracic Surgery, The University of Tokyo Graduate School of Medicine, Tokyo, Japan

**Keywords:** Annuloaortic ectasia, Marfan syndrome, Modified Ravitch, Pectus excavatum

## Abstract

**OBJECTIVES:**

This case series aimed to determine the feasibility of simultaneous modified Ravitch and David procedures for Marfan syndrome patients with pectus excavatum and annuloaortic ectasia.

**METHODS:**

Between March 2014 and December 2019, 7 consecutive patients underwent simultaneous surgery of modified Ravitch and David procedures for pectus excavatum and annuloaortic ectasia. The completion of cardiac surgery and sternal closure were followed by the modified Ravitch procedure. The bilateral fourth to seventh costal cartilages were resected, the sternal body partially wedge resected and the sternum raised anteriorly with re-suture. An oblique incision was performed on bilateral third costal cartilages, and they were fixed on top of each other, with the medial end superior and the lateral end inferior. The sternum was raised anteriorly, bypassing the fourth to seventh rib ends through the back of the sternum with threads. The feasibility and safety of the procedure were assessed through a retrospective review of the patients’ clinical charts.

**RESULTS:**

The total sample had a median age of 28 years and comprised 5 males and 2 females. There was a significant difference in the preoperative and postoperative median Haller index, which were 6.8 and 3.9, respectively. All patients were discharged without serious complications, and there was no significant recurrence of pectus excavatum at 35–92 months postoperatively.

**CONCLUSIONS:**

The results of our case series suggest the feasibility of one-stage surgery for pectus excavatum combined with cardiac surgery using the modified Ravitch procedure. Future efforts should be tailored for more uneventful postoperative clinical courses.

**Figure:**
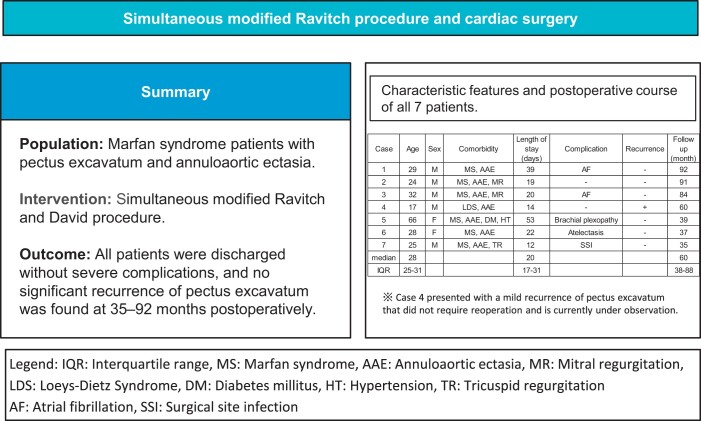

## INTRODUCTION

Pectus excavatum is one of the most common deformities of the chest wall. Pectus excavatum can cause scoliosis, heart displacement and lung volume reduction, leading to chest pain, fatigue, dyspnoea on exertion, respiratory infections and cardiac abnormalities [1]. Thus, various treatment methods for this disease have been reported in the past.

In Marfan syndrome, chest wall deformities such as pectus excavatum can be accompanied by heart disease. Pectus excavatum obstructs the surgical field because of abnormal deviation and rotation of the mediastinum. One- or two-stage repair of pectus excavatum and cardiovascular diseases has been reported in the literature [2–5].

In this case series, we report our experience concerning sternal elevation by simultaneous modified Ravitch procedure and David procedure for pectus excavatum and annuloaortic ectasia associated with Marfan syndrome.

## PATIENTS AND METHODS

### Ethics statement

For this retrospective study, informed consent was obtained in the form of an opt-out clause on our hospital’s website (https://cts.m.u-tokyo.ac.jp/thoracic-surgery/clinical_study/). This study was approved by the Institutional Review Board of The University of Tokyo Hospital (approval no. 2406).

### Study design and patients

We retrospectively collected and analysed data on patients who underwent simultaneous modified Ravitch procedure and David procedure for pectus excavatum and annuloaortic ectasia associated with Marfan syndrome and Loeys–Dietz syndrome in our department between March 2014 and December 2019.

Patient characteristics, perioperative factors and discharge status were collected from medical records. Patient characteristics included age, sex, height, weight, body mass index and comorbidities. Perioperative factors included surgical procedure (with or without resection of costal cartilage, refixation of ribs and wedge resection of the sternal body), operation time, blood loss, length of hospital stay and complications. The treatment plan was discussed for each case at a joint preoperative conference of the Departments of Cardiac Surgery and Thoracic Surgery.

All patients underwent computed tomography (CT) scans preoperatively and at least 1 month after the surgery. Preoperative and postoperative Haller indexes were calculated. The Haller index is defined as the ratio of the transverse diameter (the horizontal distance of the inside of the rib cage) and the anteroposterior diameter (the shortest distance between the frontal side of the vertebrae and the posterior surface of the sternum) at the deepest part of the pectus excavatum deformities on CT [6].

Preoperative and postoperative lung volumes were measured using a 3D image analysis system volume analyser (Synapse VincentⓇ, Fuji-Film, Tokyo, Japan) in each patient. Changes in lung volumes before and after repair of the pectus excavatum by modified Ravitch procedure were evaluated using 3DCT.

Transthoracic echocardiography was performed preoperatively and postoperatively in each patient to evaluate cardiac function before and after surgery by measuring left ventricular ejection fraction (LVEF).

### Surgical procedure

**Figure 1:**
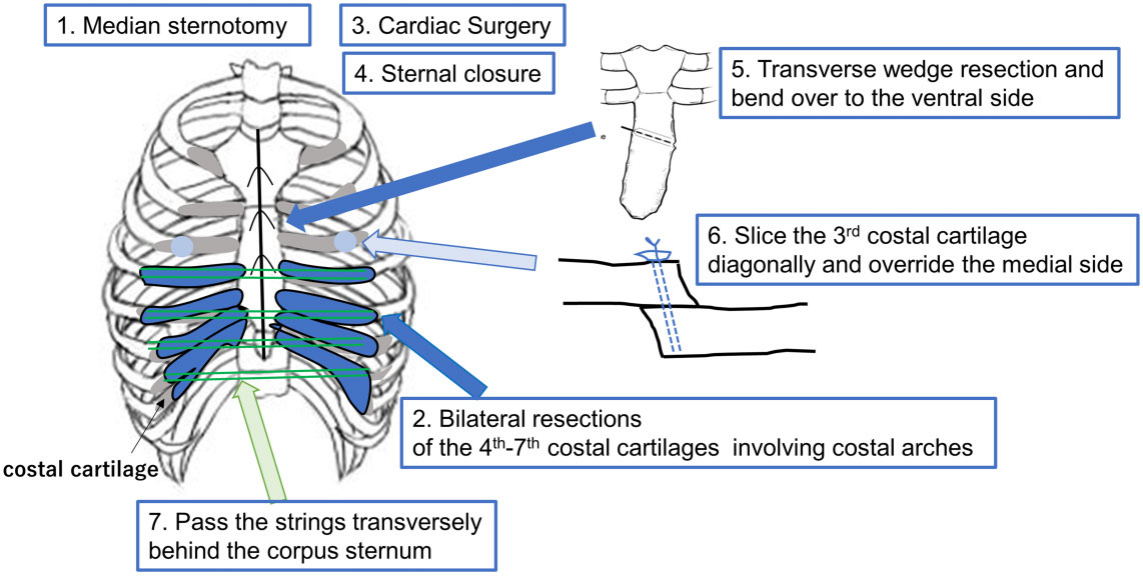
Schema of the modified Ravitch procedure. The surgery was initiated by the cardiovascular surgeon with a median sternotomy, and after cardiac surgery and sternal closure, a modified Ravitch procedure was performed. The bilateral 4th–7th costal cartilages were resected, including costal arches, and the sternal body was transversely wedge resected and bent over to the ventral side. The third costal cartilage was sliced diagonally and medial side was overlaid on top. The sternum was lifted anteriorly and the ends of the 4th–7th ribs were bypassed through the back of the sternum using strings.

Figure 1 summarizes the schema of our modified Ravitch procedure. Surgery was initiated with a median sternotomy, and the David procedure for annuloaortic ectasia was performed by the cardiovascular surgeon. The completion of cardiac surgery and sternal closure were followed by the modified Ravitch procedure. The bilateral 4th–7th costal cartilages were resected, including costal arches, and the sternal body was partially wedge resected laterally at the level of the second intercostal space. The sternum was then re-sutured so that the lower sternal body part was formed to face anteriorly. An oblique incision was placed on the bilateral third ribs, and the third costal cartilage was fixed on top of each other with the medial end superior and the lateral end inferior. After detaching the back of the sternum to avoid damaging the pleura, suture threads were placed on both sides of the cut ends of the 4th to 7th ribs or rib cartilage and were bridged with forceps and tied together. The sternum was raised anteriorly, bypassing the 4th–7th rib ends through the back of the sternum with threads.

Ravitch’s original procedure was to free the depressed sternum from the surrounding tissue, bending it anteriorly by wedge resection of the anterior and posterior surfaces of the sternum, and elevating it by overlapping the second rib cartilages on each side through an oblique incision. In our technique, only the anterior surface of the sternum was wedge resected, and the bilateral 4th–7th costal cartilages were resected up to the costal arch. The bilateral third rib cartilages were superimposed through an oblique incision, and the bilateral 4th–7th rib segments were bridged at the back of the sternum with non-absorbable polyester braided sutures to allow support of the caudal sternum.

### Statistical analyses

Descriptive statistics are summarized as medians [interquartile ranges (IQRs)] for continuous data and counts (percentages) for categorical variables. Variables were compared and analysed using paired *t*-test. *P*-values <0.05 indicated statistical significance. All statistical analyses were performed using EZR (Saitama Medical Center, Jichi Medical University, Saitama, Japan), a graphical user interface for R (The R Foundation for Statistical Computing, Vienna, Austria, version 4.2.0) [7].

## RESULTS

From March 2014 to December 2019, 7 patients underwent simultaneous modified Ravitch procedure and David procedure for pectus excavatum and annuloaortic ectasia associated with Marfan syndrome. The main characteristics of these 7 patients are summarized in Table 1. The median age was 28 years (IQR 25–31 years). There were 5 males and 2 females. The median body mass index was 19.7 kg/m^2^ (IQR 15.5–22.8 kg/m^2^), based on the World Health Organization classification, with the males categorized as underweight to normal range, and the females as pre-obese.

**Table 1:** Characteristic features of the patients (*n* = 7) in the case series

Case	Age	Sex	Height	Weight	Comorbidity
1	29	M	187	72	MS, AAE
2	24	M	182	48	MS, AAE, MR
3	32	M	186	46	MS, AAE, MR
4	17	M	164	53	LDS, AAE
5	66	F	160	64	MS, AAE, DM, HT
6	28	F	160	66	MS, AAE
7	25	M	191	60	MS, AAE, TR
Median	28		182	60	
IQR	25–31		162–187	51–65	

AAE: annuloaortic ectasia; DM: diabetes mellitus; HT: hypertension; IQR: interquartile range; LDS: Loeys–Dietz syndrome; MS: Marfan syndrome; MR: mitral regurgitation; TR: tricuspid regurgitation.

The details of the surgeries and the postoperative course of the patients are shown in Table 2. Costal cartilage resection was performed in all cases, refixation of ribs was performed in 4 cases, wedge resection of the sternal body was performed in 4 cases and sternal elevation with suture threads was performed in 6 cases. Non-absorbable sutures were used in all cases. Thick sutures ranging from size 3 to finer sutures to 2–0 were selected depending in the case.

**Table 2:** Details of the surgeries and postoperative course for the patients (*n* = 7) in the case series

Case	Resection of costal cartilage	Refixation of ribs	Wedge resection of the sternal body	Sternum elevation	Suture thread	Operation time (min)	Thoracic Surgery (min)	Blood loss (ml)	LOS (days)	Complication	Recurrence	Follow-up (month)
1	О	О	О	О	NA	713	104	1335	39	AF	–	92
2	О	О	О	О	NA	591	146	410	19	–	–	91
3	О	О	О	О	NA	805	214	1270	20	AF	–	84
4	О	О	О	–	NA	534	152	210	14	–	+	60
5	О	–	–	О	NA	625	185	780	53	Brachial plexopathy	–	39
6	О	–	–	О	NA	462	110	450	22	Atelectasis	–	37
7	О	–	–	О	NA	519	73	810	12	SSI	–	35
Median						591	146	780	20			60
IQR						530–669	128–169	128–169	17–31			38–88

AF: atrial fibrillation; IQR: interquartile range; LOS: length of hospital stay; NA: non-absorbable suture; SSI: surgical site infection.

In 2 cases, due to the extremely narrow chest cavity, costal cartilage resection was performed before the median sternotomy to obtain an adequate operative field and mobility of the inferior end of the sternum and attached medial costal fragments. This manoeuvre also eliminated the risk of cardiac compression by opening the chest.

The median operative time was 591 min (IQR 530–669 min), including the David procedure; the median blood loss was 780 ml (IQR 430–1040 ml); and the median hospital stay was 20 days (IQR 17–31 days). There was no mortality recorded.

Complications were observed in 5 patients. Cases 1 and 3 required electrical cardioversion for atrial fibrillation. Case 5 showed postoperative brachial palsy, but the patient's symptoms tended to improve conservatively. Case 6 had postoperative respiratory failure due to atelectasis, which improved with the use of a non-invasive ventilator. Case 7 developed a postoperative infection of the median sternotomy wound that healed with negative pressure wound therapy.

**Figure 2:**
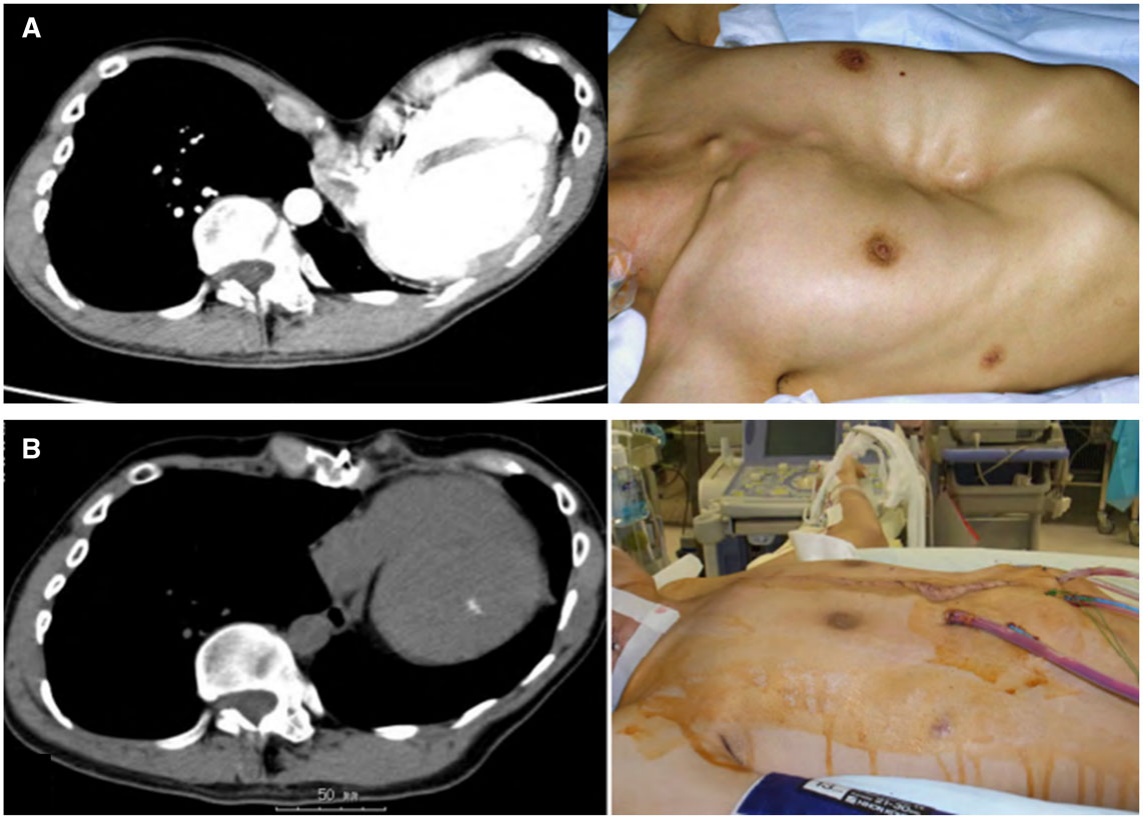
Computed tomography and picture (**A**) before and (**B**) after the surgical procedure. The morphology of the chest wall improved both visually and on computed tomography images before and after the surgery.

Figure 2 shows pre- and postoperative CT images and the morphology of the chest wall. All patients were discharged from the hospital. There were no significant recurrences of pectus excavatum at 35–92 months postoperatively, but case 4 presented with a mild recurrence of pectus excavatum that did not require reoperation and is currently under observation. There was a significant difference between the median Haller index, at 6.8 (IQR 5.8–13.7) preoperatively and 3.9 (IQR 3.5–4.2) postoperatively (*P* < 0.05).

Changes in lung volumes were measured in 6 cases. Case 4 was excluded because lung volumes could not be measured from CT due to inappropriate imaging conditions. CT scans were performed preoperatively, within 6 months and after 1 year postoperatively (only that of case 7 was performed 1 month postoperatively). The median preoperative lung capacity was 3705 ml (IQR 2395–4576 ml) and the median postoperative lung capacity was 3805 ml (IQR 2516–4161 ml). Contrary to expectations, postoperative lung volumes decreased in 4 patients and increased in 2 patients, but overall, they did not change significantly (*P* = 0.69).

Changes in LVEF were measured in all patients. Transthoracic echocardiography was performed within 1 year pre- and postoperatively. There was no significant difference in the median LVEF, at 64% (IQR 60–69%) preoperatively and 66% (IQR 63–66%) postoperatively (*P* = 0.44).

## DISCUSSION

The first technique of pectus excavatum repair was proposed by Ravitch in 1949; it was an open surgery that required partial resection of the cartilage, xiphoid excision and osteotomy of the sternum [8]. Multiple modifications to this procedure were proposed, such as the placement of a metal strut to support the sternum that would be removed after 6 months to a year. In 1998, Nuss proposed a minimally invasive technique, which uses a lateral chest wall incision, and is the most widely used surgical technique for pectus excavatum in the world today [9, 10]; however, its application in adult patients is controversial [11]. Taci *et al.* suggested that postoperative complications and recurrence rates were not statistically different between the Nuss and Ravitch procedures [12].

Several problems should be addressed in cardiac surgery associated with pectus excavatum. It is sometimes difficult to obtain an adequate operative field through routine median sternotomy because of severe posterior concavity of the sternum, which leads to the deviation of the heart and great vessels to the left hemithorax. The main issue in such cases is how to approach and repair both the pectus excavatum and any cardiac displacement into the left thoracic cavity. There have been various reports of cardiac surgery associated with pectus excavatum, including one- and two-stage surgeries. Considering the cardiac compartment syndrome due to compression from the depressed sternum after open-heart surgery, it is desirable to perform the surgery as 1 stage as possible.

It has also been reported that there is a high incidence of non-valvular arterial fibrillation associated with pectus excavatum. Furthermore, the incidence of arterial fibrillation associated with severe pectus excavatum is 5-fold higher than in patients without pectus excavatum [13]. Some reports discourage simultaneous repair of both lesions because of the potential for major complications, such as limited exposure of the heart, excessive bleeding and increased risk of wound infection [14]. However, other studies have reported successful one-stage corrections of both lesions without any complications [4, 5]. We have also successfully corrected both lesions simultaneously in a case that required median sternotomy.

The first technique of pectus excavatum repair described by Ravitch dissected the sternum and affected ribs [8]. Multiple modifications required long-term ventilator or metallic bar support of the sternum. Javangula *et al.* [15] reported the use of a ‘sternal bed’ with 2 wide strips of a Gortex patch as an alternative to a metallic bar. Kawamura *et al.* [5] reported an open door approach by partial sternotomy and sterno-costo-chondroplasty to divide and refix the right and left ribs, which was advantageous in partially dividing the sternum so the right ribs can be opened like a door to expose the deviated mediastinum. In the present case series, there was a case in which an oblique incision was difficult because the rib cartilage was fragile due to the replacement of bony tissue by age, so several options for sternal elevation should be prepared.

Despite the preferential use of the Nuss procedure for pectus excavatum due to its minimal invasiveness, the Ravitch procedure in a median sternotomy approach performed simultaneously with cardiac surgery should still be considered. We used the modified Ravitch procedure of costal cartilage resection, rib refixation, wedge resection of the sternal body or sternal elevation by suture thread, depending on the case. Kanagaratnam *et al.* [16] demonstrated that the Nuss method was reported to cause more pain, especially in adult patients, but this could not be validated due to a lack of self-reported data and data on analgesic duration. In cases of pectus excavatum where a median sternotomy is used in cardiac surgery, it seems logical to choose the modified Ravitch procedure over the Nuss technique, which may cause increased pain, and the same is true from an aesthetic standpoint.

Pulmonary function was one of the most promising outcomes of the modified Ravitch procedure for the treatment of pectus excavatum. Sakamoto *et al.* [17] demonstrated little or no improvement in pulmonary function in adolescent patients postoperatively. They concluded that the lung capacity does not change postoperatively because the thoracic spine protrudes forward as much as the sternum protrudes forward. In fact, in our present study, there was no improvement in pulmonary function after the modified Ravitch procedure in adults.

A correlation between the Haller index and cardiac function (LVEF and RVEF) has been demonstrated in adolescents around 15 years of age [18]. Moreover, improvement in cardiac function in the early postoperative period has been reported to be sustained at follow-up [19], but this is not clear in adults, as was not shown in the present study.

### Limitations

This study had some notable limitations. It was a retrospective, observational and single-institution study with a small number of cases. It was only exploratory in nature and can only provide an indication of the outcomes of the simultaneous procedure. It only included pectus excavatum patients with annuloaortic ectasia associated with Marfan syndrome and Loeys–Dietz syndrome; therefore, the results can only be applied to patients with cardiac disease.

## CONCLUSION

In conclusion, while functional improvement is not satisfied, the results of our case series suggest the feasibility of one-stage surgery for pectus excavatum combined with cardiac surgery using the modified Ravitch procedure. It is acceptable in terms of complications and recurrence, but a further accumulation of cases is needed to reduce complications.

## Data Availability

The data underlying this study will be shared on reasonable request from the corresponding author.

## Notes

The presentation was given at the 73rd Annual Scientific Meeting of the Japanese Association for Thoracic Surgery (Japan, Nagoya, October 29th to November 1st, 2020, On-Demand).

## ABBREVIATIONS

CT: Computed tomography
IQRs: Interquartile ranges
LVEF: Left ventricular ejection fraction
